# Experimental and Numerical Study of Micropitting Initiation in Real Rough Surfaces in a Micro-elastohydrodynamic Lubrication Regime

**DOI:** 10.1007/s11249-018-1110-2

**Published:** 2018-11-02

**Authors:** M. F. AL-Mayali, S. Hutt, K. J. Sharif, A. Clarke, H. P. Evans

**Affiliations:** 1 College of Engineering, University of Al-Qadisiyah, Al Diwaniyah, Iraq; 2 School of Engineering, Cardiff University, Cardiff, UK

**Keywords:** Micropitting, Contact fatigue, Gears, Micro-EHL, Mixed lubrication, Surface roughness

## Abstract

Micropitting is a form of surface fatigue damage that happens at the surface roughness scale in lubricated contacts in commonly used machine elements, such as gears and bearings. It occurs where the specific film thickness (ratio of smooth surface film thickness to composite surface roughness) is sufficiently low for the contacts to operate in the mixed lubrication regime, where the load is in part carried by direct asperity contacts. Micropitting is currently seen as a greater issue for gear designers than is regular pitting fatigue failure as the latter can be avoided by control of steel cleanliness. This paper describes the results of both theoretical and experimental studies of the onset of micropitting in test disks operated in the mixed lubrication regime. A series of twin disk mixed-lubrication experiments were performed in order to examine the evolution of micropitting damage during repeated cyclic loading of surface roughness asperities as they pass through the contact. Representative measurements of the surfaces used in the experimental work were then evaluated using a numerical model which combines a transient line contact micro-elastohydrodynamic lubrication (micro-EHL) simulation with a calculation of elastic sub-surface stresses. This model generated time-history of stresses within a block of material as it passes through the contact, based on the instantaneous surface contact pressure and traction at each point in the computing mesh at each timestep. This stress time-history was then used within a shear-strain-based fatigue model to calculate the cumulative damage experienced by the surface due to the loading sequence experienced during the experiments. The proposed micro-EHL model results and the experimental study were shown to agree well in terms of predicting the number of loading cycles that are required for the initial micropitting to occur.

## Introduction

**Fig. 1:**
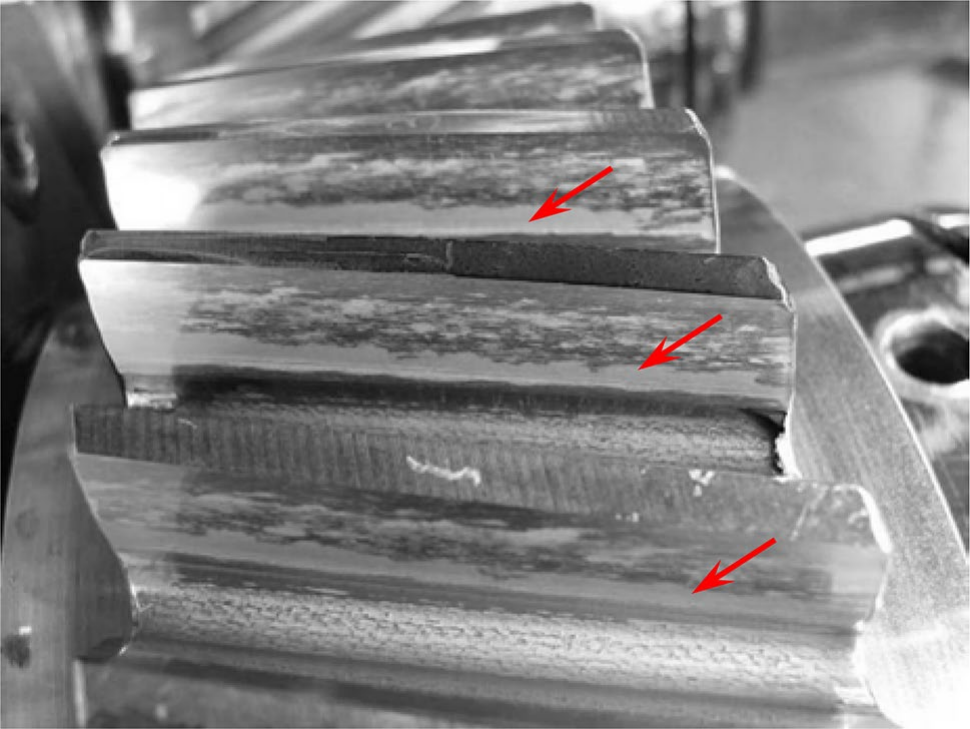
A helical gear with micropitting visible as a matted region on the dedendum of the teeth (arrowed), from [5]

Micropitting is a type of surface fatigue that is associated with roughness effects, which occurs on the working faces of gear teeth but can also occur in rolling element bearings. It is generally observed on the scale of the surface roughness asperities in heavy duty hardened steel gears [1]. Historically, there has been a tendency to regard micropitting as a secondary problem with attention being focussed on (macro) pitting caused by fatigue on the scale of the Hertzian contact. However, with improvements in steel cleanliness and processing, attention is now focussing on micropitting, particularly given recent problems with micropitting in the speed increasing gearboxes of wind turbines [2, 3]. The shallow surface cracks which occur in micropitting may branch and join, leading to larger scale macro pitting and eventual severe degradation of the involute profile and potential tooth breakage [4]. An example of micropitting may be seen in Fig. 1, which is taken from a micropitting test at Newcastle University [5]. Here, the micropitting may be observed in the dedenda of the teeth, where it commonly occurs, and is characterised by a frosted or matted appearance in comparison to regions without damage.

**Fig. 2:**
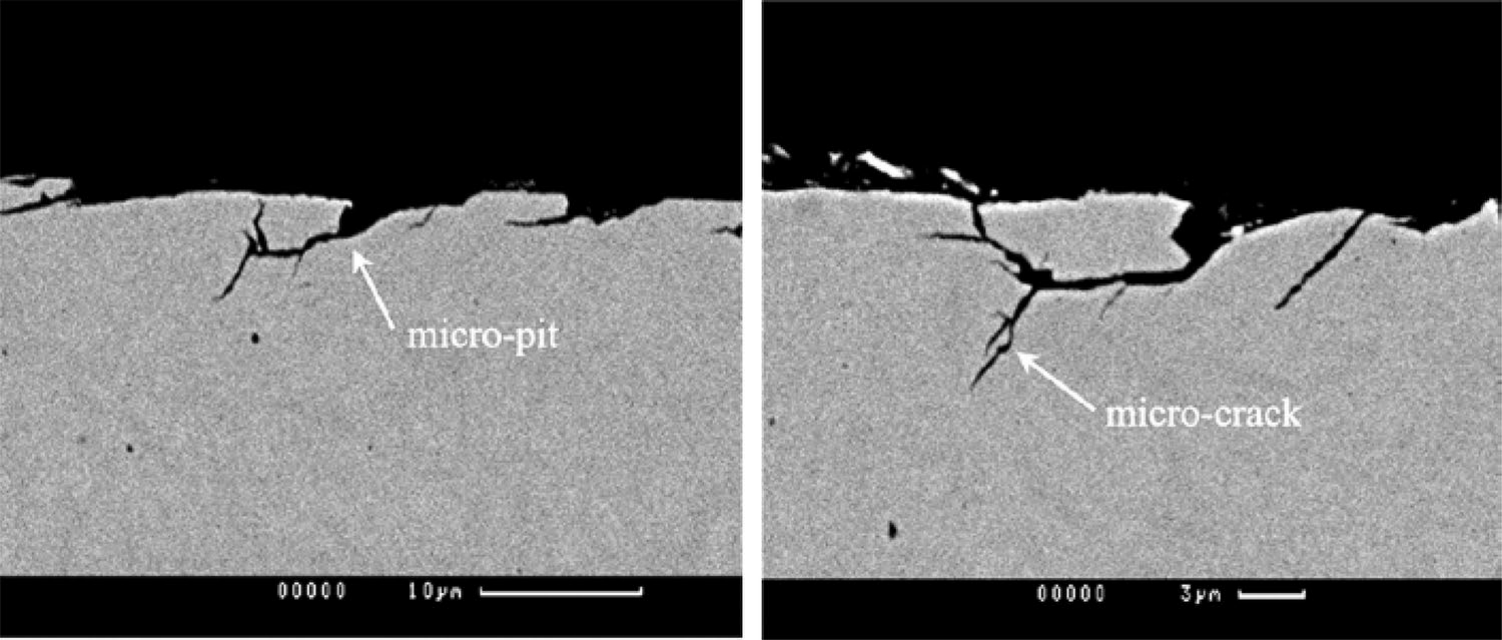
Sections through micropits showing crack growth, from [5]

The propagation of micropitting is also associated with the crack pressurisation phenomena [6]. Furthermore, the cracks propagate in different directions on either side of the pitch line, where the sliding direction is reversed [7]. Micropitting is on a much smaller scale than general pitting, with pits measured in microns and often associated with plastic deformation due to the running in process [8, 9]. Micropits are typically 10–30 µm in diameter and can be up to 10 µm deep, as can be seen in the fatigue damaged metallurgical sections of the gear surface teeth in Fig. 2. In the current paper, the observed micropits are shallower and about 2 µm deep. This observation is compatible with Fig. 2, where the region of the micropits measurable by stylus profilometry are around 2–3 µm deep. These cracks occur at the tips of the asperities and extend at a shallow angle (typically 10–30°) to the surface, opposite to the direction in which sliding frictional traction acts on the surface [7, 9].

Presently, the mechanisms of micropitting initiation and propagation are not well understood but it is believed to be a fatigue related phenomenon. To understand gear life, it is necessary to analyse the micropitting mechanism in more detail so as to understand the main factors that affect performance so as to improve the reliability of gears. These factors include asperity contact pressure, surface traction, and subsurface stress under elastohydrodynamic lubrication (EHL) conditions. Transient effects occur in gears due to the motion of surface roughness relative to the contact. These relative velocities are different for the two surfaces according to the distance from the pitch point. This means that surface asperities on the tooth surfaces slide past with each other, generating highly transient localised pressures during their interaction that are of the order of the surface hardness (flow pressure) and well in excess of the dry Hertzian smooth surface pressure.

An effective micro-elastohydrodynamic lubrication (micro-EHL) simulation model must include the following features: low specific film thicknesses or *Λ* ratios (the ratio of calculated smooth-surface nominal lubricant film thickness to composite surface roughness); highly transient effects caused by moving roughness through the EHL contact region; and non-Newtonian fluid behaviour, which can be caused by high shear rates. Under these severe conditions, numerical EHL solvers are required that can deal with thin film/high roughness situations, particularly under conditions where there may be a degree of mixed lubrication in which load is shared between interacting asperities and thin lubricant films.

The simulation of mixed lubrication contacts has been the subject of much research, from early work using sinusoidal roughness features and non-Newtonian lubricant treatments in transient micro-EHL models [10, 11], to more recent developments which feature real, measured rough surfaces [12], applied under realistic conditions representative of gear contacts [13], in three-dimensional point contacts [14], and in more heavily mixed and even boundary lubrication conditions [15].

Compared to the body of research into micro-EHL, there has been little work carried out to link the results of these simulations with fatigue-related failure of the rough surfaces in lubricated contacts. Hannes and Alfredsson [16] investigated rolling contact fatigue using idealised asperity geometries in a dry-contact stress calculation. They linked this with a crack growth model to investigate the effects of surface finish on surface-induced contact fatigue. Pu *et al*. [17] again used idealised asperity geometries, with sinusoidal surfaces used in mixed-EHL simulations coupled with a fatigue model, based on von-Mises stress distributions calculated from the EHL results. Noting the absence of known material data for the surface layers of typical heat treated steel components, they considered the relative rather than absolute fatigue performance of a range of surface geometries and operating conditions. Other workers [18] have considered the development of micropitting using a fracture-mechanics approach to study crack propagation during micropitting, and applied the developed model to a finite element-based stress-analysis of a spur gear pair. However, this model did not directly take into account surface roughness effects.

A realistic model of lubricated real rough surfaces requires a solution to the micro-EHL problem, coupled with an appropriate fatigue model. Li and Kahraman [19] proposed a model which used the boundary element method to calculate stresses based on surface pressures and tractions from a mixed-EHL model. These stresses were then used in a fatigue model using a multi-axial fatigue criterion based on a characteristic plane. Example results were given for a ball-on-disk contact and in [20] for a two-disk test rig, with comparison between predictions and experimental results highlighting areas of potential improvement for the model. Brandao *et al*. developed a surface contact model for gear teeth [21], and later a combined model which took account of both wear and surface contact fatigue [22]. In their work, the mixed lubrication pressure and traction distributions were calculated by interpolation between the smooth surface (Hertzian) pressure distribution and the dry contact problem. They then calculated sub-surface stress distributions and adopted the Dang-Vang multi-axial high-cycle fatigue criterion to predict micropitting in spur gear tooth flanks. They compared their results to experiments, and found qualitative agreement.

Previous work by researchers at Cardiff has developed micro-EHL-based fatigue models for the contact of real rough surfaces under mixed lubrication conditions [23]. These models have been previously used to compare the predictions of various fatigue criteria used to assess fatigue performance [24], or to make qualitative comparison with micropitting tests [5].

In the current paper, these simulation methods have been employed to solve the problem of micro-EHL in test disks, which were used as part of an experimental study of micropitting. The results of micro-EHL analysis (i.e. transient surface pressure and shear traction distributions) have then been used to provide the surface loading experienced by the moving material of the counterfaces in order to determine the stress history experienced in passing through the EHL contact. The stress history is then post-processed to investigate the fatigue damage accumulation of the disks at and immediately below the contacting surfaces. The micro-EHL analysis includes conditions where there is significant interaction of the asperity features on two rough surfaces. Using the real, measured surface roughness profiles under the real EHL operating conditions is judged to be an important requirement to quantify the surface fatigue lives because surface profile topography is significantly modified by running-in.

## Mixed-EHL Model and Material Fatigue Theory

The fatigue damage model depends on the results of the transient micro-EHL solutions for the surfaces in rolling/sliding contact. The micro-EHL model employed in this work has been described in detail elsewhere [14], and is outlined here for completeness. Essentially, the two fundamental equations that define the EHL problem (Reynolds equation, incorporating non-Newtonian effects, and the elastic deflection equation) are fully coupled in the numerical analysis scheme, using the differential deflection form of the elastic deflection equation.

Using representative sections of run-in surfaces measured during the experimental work, of twice the Hertzian contact dimension (2*a*), longer multiprofiles are created by repeating the individual profiles (head to tail) with the joins between the repeated representative profiles made at deep valley features to ensure that the extended profile does not introduce any prominent new asperity features artificially. The purpose of using multiprofiles is to be able to consider a wide range of the asperity interactions for the two surfaces during the micro-EHL contact simulation. The transient analysis output starts from the corresponding smooth surface steady state solution as an initial condition by calculating the pressure distribution and film thickness. Once the steady state solution has been obtained, it is used as the starting condition for the transient analysis and the two rough surface profiles are then fed into the contact from the inlet boundary time step by time step in accordance with the surface kinematics. For each time step, a converged solution of the pressure and film thickness with the current surface geometry is obtained. The lubricant pressure and film thickness values for the previous time step are used as starting values for the next time step during the solution process. The model is robust under heavily mixed lubrication conditions, including occurrences of dry contact between asperities as described in [14].

The results from EHL analysis provide the time-dependent pressure and film thickness over the solution region which extends from *x*/*a* = − 2.5 to *x*/*a* = 1.5. The EHL model was analysed with a grid dimension Δ*x* = *a*/200, and a time step Δ*t* chosen such that the faster moving surface passed through a distance 0.5 Δ*x* in each time step. Introducing the fast surface roughness profile into the solution zone is delayed to make both roughness profiles reach the exit at the same time, and at that stage the analysis becomes ‘fully rough’. As the asperities pass through the EHL contact zone, they interact with asperities on the counter surface, which are moving through the contact at a different speed. This increases the probability of the fatigue damage at the scale of the surface roughness due to the resulting cyclic loading of the asperities.

**Fig. 3:**
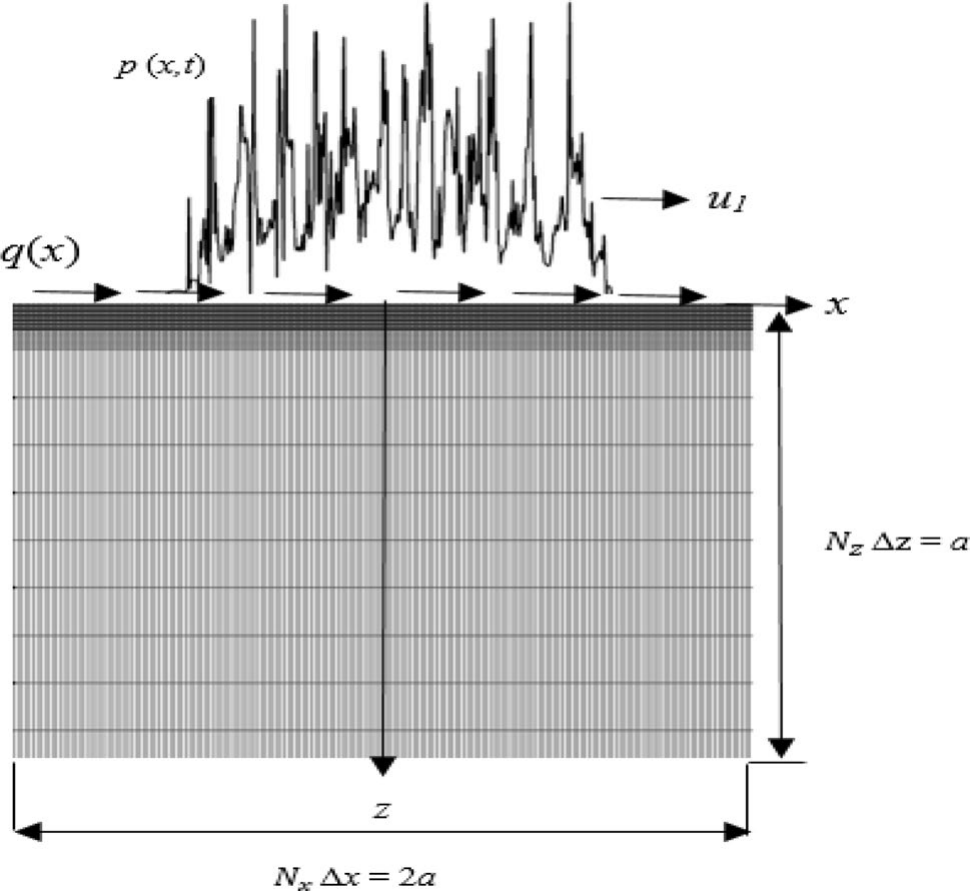
Schematic illustration of the computational domain and grid mesh used

The mixed EHL time step results are used to calculate the stress history at the lubricant/solid interface and to analyse a block of the near surface material as it passes through the contact region. At each time step in the EHL analysis, stored values of pressure, film thickness and surface traction are used to determine subsurface stress and strain history at each point in the representative block of material. The calculation of the stress and strain distributions for plane strain are determined by convolution integrals, using equations of the form:

1 $$\sigma (x,z,t)=\int_{{{x_1}}}^{{{x_2}}} {p(s,t)F(x - s,z)ds} +\int_{{{x_1}}}^{{{x_2}}} {q(s,t)G(x - s,z)ds}$$

for each of the stress components *σ*_*xx*_ (*x,z,t*), *σ*_*zz*_ (*x,z,t*) and *τ*_*xz*_ (*x,z,t*), where *x* is the tangential co-ordinate, *z* is the normal co-ordinate, and *t* is time, the weighting functions *F* and *G* for the stress component considered are given in [25]. This evaluation of the instantaneous elastic stress distribution is applied to rectangular trial blocks of selected material whose dimensions are chosen to be 2*a* parallel to the rough surface and *a* perpendicular to the surface, as shown in Fig. 3, where a is the Hertzian semi-contact width. The computational domain for stress and fatigue analysis is discretised into *Nx* × *Nz* grid elements. The grid increment *Δz* is variable with the finest mesh used near the surface to capture the possible high stress gradients due to the surface roughness. The *Δx* increment is kept uniform and sufficiently small (on the order of micrometres) to capture stress concentrations on the scale of the surface roughness. The evaluation of stresses using this approach assumes that the stress field remains elastic (or experiences elastic shakedown when a material is loaded repeatedly if the contact pressure does not exceed the shakedown limit, as evidenced by the rapid and stable running-in observed in such experiments [8]).

The multi-axial stress components are then used for fatigue damage assessment using a cumulative damage model. This involves three phases: (a) finding the stress and strain levels for each effective loading cycle, (b) estimating the damage for each effective loading cycle using an appropriate fatigue model, and (c) accumulating the damage for each effective loading cycle to determine the (calculated) damage experienced by material over the loading sequence using the Palmgren–Miner cumulative damage theory.

The rainflow counting method [26] is adopted to calculate the effective load cycles and the corresponding stress levels for each such cycle. These cycles correspond to strain hysteresis loops, which are determined from the strain history. Numerous different models are available for predicting the fatigue damage for each effective loading cycle and the difference in their damage predictions is considered in [24]. For the current paper, the shear strain model proposed by Fatemi and Socie [27] is used to predict the variable amplitude multiaxial fatigue damage and is expressed by Eq. (2), which gives the number of loading cycles to failure ${N}_{f}$ for the effective loading cycle.

2 $$\frac{{\Delta {\gamma _{\hbox{max} }}}}{2}\left( {1+k\frac{{\sigma _{n}^{{\hbox{max} }}}}{{{\sigma _y}}}} \right)=\frac{{{{\tau ^{\prime}}_f}}}{G}{\left( {2{N_f}} \right)^b}+{\gamma ^{\prime}_f}{\left( {2{N_f}} \right)^c}$$

where ${{\Delta {\gamma _{\hbox{max} }}} \mathord{\left/ {\vphantom {{\Delta {\gamma _{\hbox{max} }}} 2}} \right. \kern-0pt} 2}$ refers to the amplitude of shear strain on the test plane considered, and $\sigma _{n}^{{\hbox{max} }}$ refers to the maximum tensile stress which is normal to the plane for the effective loading cycle. The values of the material parameters used for fatigue damage modelling are given in Table 1 and correspond to those for SAE4340 steel, as given in [28]. The damage sustained in one effective load cycle is $F_{f}^{{ - 1}}$ and the total damage, *D*, is the sum of the damage for each effective load cycle as the material traverses the EHL contact area according to the Palmgren–Miner damage accumulation rule.

**Table 1:** Eq. (2) parameter values

K	Material constant	1.0
*G*	Shear modulus (GPa)	80
${\sigma _y}$	Yield strength for the cyclic stress–strain curve (GPa)	0.827
${\tau ^{\prime}_f}$	Shear fatigue strength coefficient (GPa)	1.15
${\gamma ^{\prime}_f}$	Shear fatigue ductility coefficient	0.831
*b*	Fatigue strength exponent	− 0.091
*c*	Fatigue ductility exponent	− 0.6

The parameters values of ${\tau ^{\prime}_f}$ and ${\gamma ^{\prime}_f}$ were estimated from the fatigue strength coefficient ${\sigma ^{\prime}_f}$ = 2 GPa and the fatigue ductility coefficient ${\varepsilon ^{\prime}_f}$ = 0.48 [28] for pure shear conditions as ${{{{\varepsilon ^{\prime}}_f}} \mathord{\left/ {\vphantom {{{{\varepsilon ^{\prime}}_f}} {\sqrt 3 }}} \right. \kern-0pt} {\sqrt 3 }}$ and $\sqrt 3 {\varepsilon ^{\prime}_f}$, respectively, Dowling [29]

3 $$D=\sum\limits_{{{\text{all}}\,{\text{effective}}\,{\text{loading}}\,{\text{cycles}}}} {N_{f}^{{ - 1}}}$$

A damage value *D* = 1, corresponds to predicted fatigue failure. The model is applied over the material block considered at all mesh points and at each of these points, it is applied to test planes of all orientations in turn. The maximum value of *D* obtained at a point when all orientations are considered is the damage calculated to occur at that point and the corresponding plane is the critical plane for that point. The fatigue life at any point in the component material blocks is the reciprocal of *D*. This is the number of traverses through the EHL contact zone associated with fatigue at that point (the number of gear meshing cycles in a gear contact analysis).

The fatigue model described here was applied to a series of micropitting experiments which are described in the next section.

## Micropitting Experiments

Tests were conducted using a power-recirculating twin disk test rig which is designed to simulate the contact between heavily loaded gears with simplified kinematics. It has been used by researchers to study surface wear mechanisms, such as running-in and micro-pitting [8], and novel measurement techniques for mixed lubrication, such as contact resistance [30] and, most recently, acoustic emission.

**Fig. 4:**
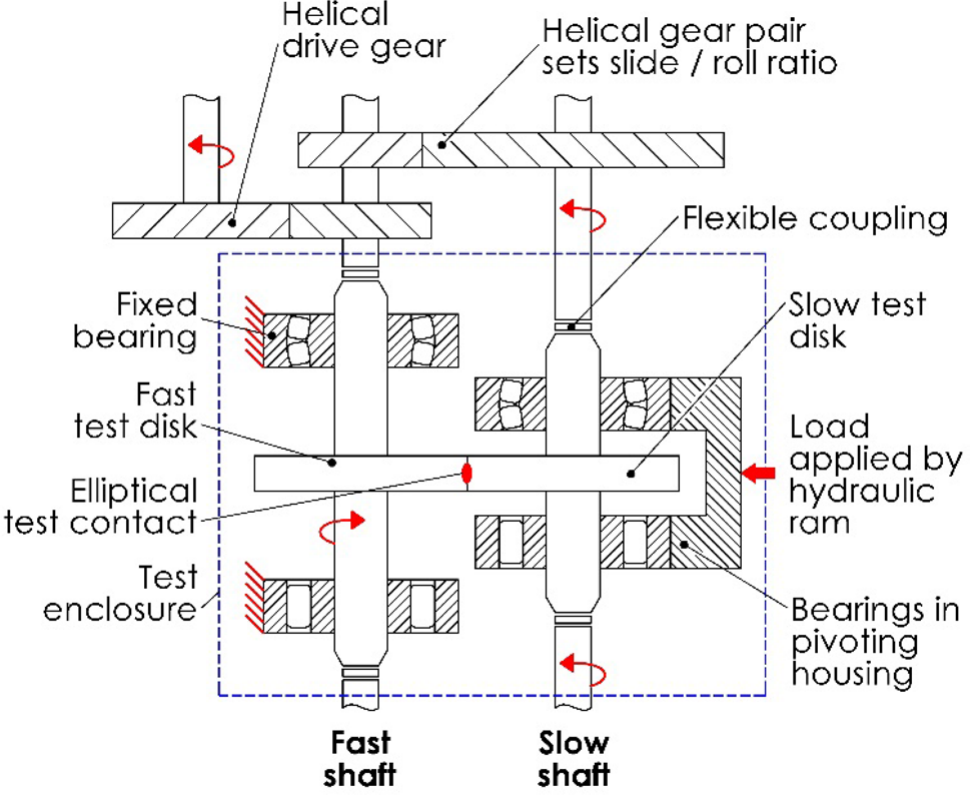
Schematic of the main components of the twin disk rig

Figure 4 shows a schematic of the main components of the rig, which is described in detail in [8, 30]. The test contact is made between two geometrically identical disks driven by a helical gear pair, the ratio of which determines the slide / roll ratio (SRR) of the contact. A constant SRR, as generated by this rig, differs from that of meshing gear teeth, where it varies over the tooth contact cycle; however, it significantly reduces the complexity of the measurement and analysis techniques required to study the contact.

The disks are made of a case hardened gear steel meeting Rolls-Royce specification 6010. Each disk is 76.2 mm in diameter and is crowned to a radius of 304.8 mm, producing a spheroidal contact face. This results in an elliptical contact having a 4:1 aspect ratio with the minor axis aligned in the rolling / sliding direction. The disk crown is generated using a conical grinding wheel that results in a surface lay with the peaks and valleys aligned nearly normally to the slide / roll direction. This replicates the main characteristics of the roughness orientation found in ground gear tooth contacts.

**Fig. 5:**
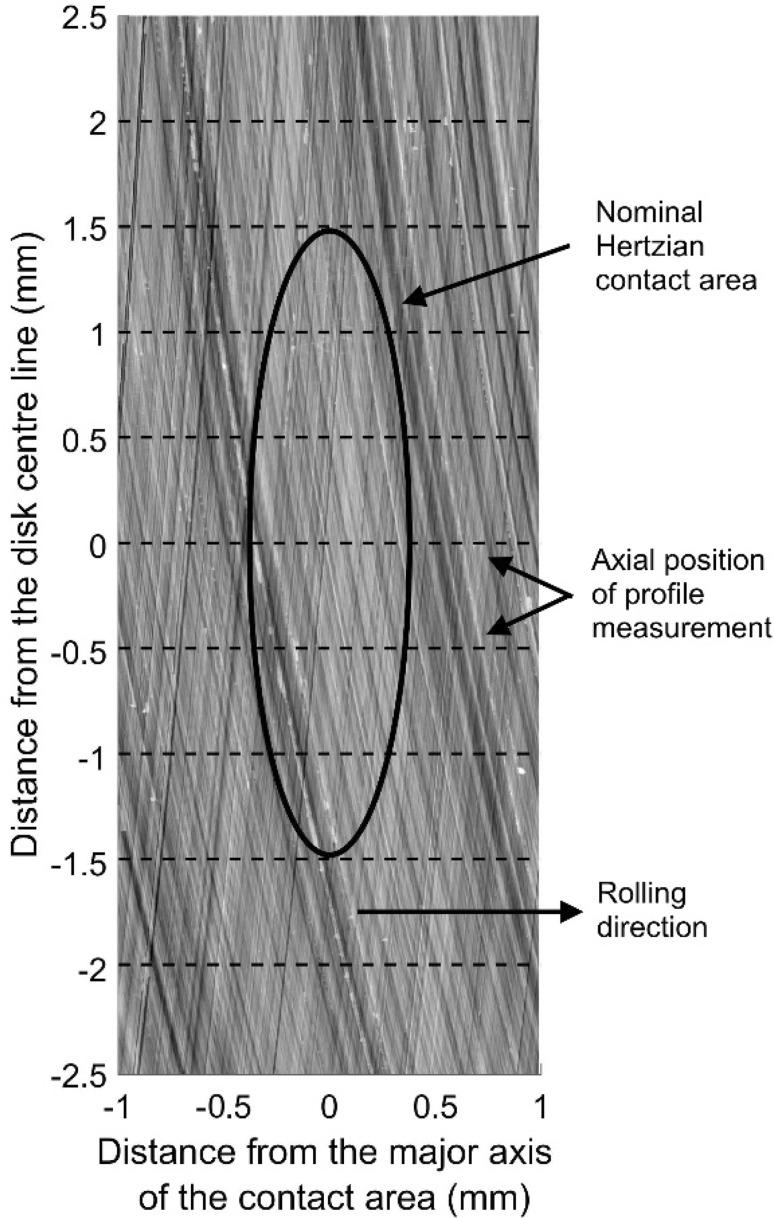
Composite surface roughness of the disks prior to testing, valleys are dark, peaks are light. Also shown are the nominal Hertzian contact area for the test load and the axial positions of the profile measurements

Three dimensional surface height measurements of the disks used in this paper were taken before and after testing using a Taylor Hobson profilometer. Figure 5 shows their pre-test composite (sum) roughness as orientated in the contact. It can be seen that grinding process produces a roughness profile that is ‘extruded’ across the disk width along a slightly curved path. The disks were orientated in the rig such that the resulting roughness features cross at a shallow angle producing a cross-hatched composite roughness which is a feature of the contact roughness of helical form ground gears [31]. The Vickers hardness and initial surface roughness of the disks used for this work were measured. Both disks had a surface hardness of 760 Hv, with initial R_q_ of 0.42 µm for the fast disk and 0.48 µm for the slow disk.

The disks are lubricated by an extreme pressure gear oil conforming to Defence Standard OEP-80 [32]. The particular formulation used is mineral-based, and was chosen for continuity with previous tests conducted by the authors under similar mixed lubrication conditions, e.g. [8, 30]. The oil is pumped from a heated bath and sprayed onto the inlet and outlet of the disk contact, it is also used to lubricate the gears and bearings of the rig. The heated oil increases the ambient temperature in the test enclosure, and testing is started only after it has reached thermal equilibrium. The temperature of each disk is measured using an embedded thermocouple located 3 mm below the contact surface and on the centre line.

The test procedure was designed to induce micro-pitting fatigue using as few variables as possible in order to simplify the fatigue analysis. Thus the slide/roll ratio, load, oil temperature and speed were kept constant for the test. Their values and the resulting contact parameters are shown in Table 2.

**Table 2:** Rig and contact parameters

Slide/roll ratio	0.5
Fast disk speed (rpm)	1000
Entrainment velocity (m/s)	3.2
Load (N)	1460
Maximum Hertzian contact pressure (GPa)	1.2
Hertzian contact dimensions (mm)	0.8 × 3.0
Oil bath temperature (°C)	80

New test disks were used for this experiment meaning there was an initial, relatively short, running-in period before the surface roughness became quasi-stable. The test was paused periodically to make surface measurements in order to validate the end of the running-in period and detect the start of micro-pitting fatigue. The period of fatigue testing in-between each surface measurement is referred to as a load stage. The initial load stages were of nominally 3000 fast disk rotational cycles in duration (load stages 1 and 2), with subsequent load stages being of approximately 100,000 fast disk cycles in duration.

At the start of each load stage, the rig was run at the test speed with the disks unloaded and not in contact until thermal equilibrium was reached. Upon loading, there was a short period of increasing disk temperature due to frictional heating at the contact before a new thermal equilibrium was reached. This temperature change was unavoidable but of negligible duration, given the length of each load stage. At the end of each load stage, the rig was allowed to cool overnight before surface measurements were taken in order to avoid introducing error due to thermal expansion.

The rig is designed to allow in situ profile measurements of both disk surfaces using a portable Taylor Hobson 2D profilometer. This is an important feature for this work as it allows changes in the disks’ surface roughness to be measured over a series of tests whilst preserving their precise orientation. The profiles are measured in a circumferential direction and the profilometer mounted on an axially aligned linear stage. Repeat position profile measurements are made as follows: A score mark on the side of the disk is used as a coarse reference point for the circumferential position of the profile scan. A precise reference is not required as cross-correlation techniques are used to accurately re-align the profiles after measurement. The axial position is set using the edge of the disk as a reference point. With the profilometer stylus initially positioned on the approximate centre line of the disk, it is slowly moved outwards using the linear stage until the surface height drops by 60 µm indicating that the edge of the disk has been reached by the stylus. This axial position, which is measured by dial gauge, is then used as the reference point by which the precise axial measurement positions are then set. The error in axial positioning is likely to be at least ± 5 µm, given the resolution of the dial gauge.

For this experiment, profile measurements 12 mm in length were taken, from both disks, at a single circumferential position. Nine axial profiles were taken, at the disk centre line and spaced at 0.5 mm increments from the centre line as shown in Fig. 5. The Hertzian contact area for the test load is shown with its minor axis nominally aligned with the disk centre-line. The range of axial positions was chosen to ensure the whole width of the contact was covered with some profiles located outside the contact to use for comparison. The roughness was extracted from the profiles using a Gaussian filter with a cut-off of 0.8 mm.

**Fig. 6:**
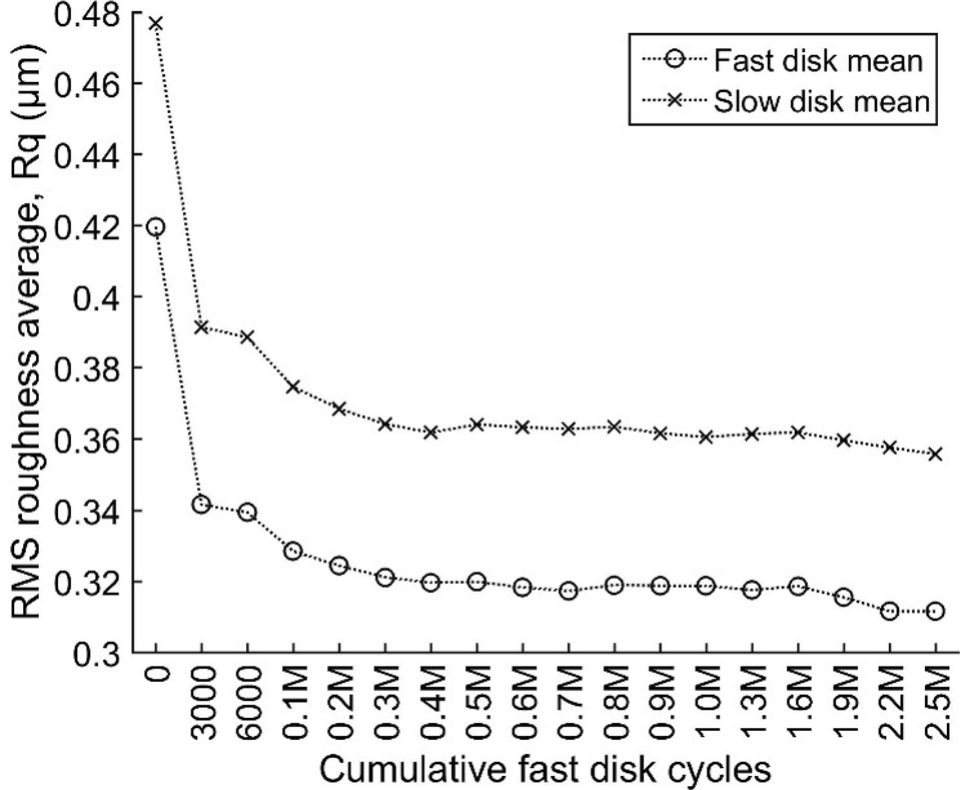
The RMS roughness, Rq, from the contact area of each disk versus the cumulative number of fast disk cycles. Each roughness value is calculated as the mean of the five profiles with axial positions within the contact (see Fig. 5)

Figure 6 shows points when the test was paused and roughness measurements were taken, in terms of cumulative fast disk cycles. As expected, a relatively large decrease in the R_q_ roughness of both disks occurred within the first 3000 fast disk cycles due to running-in, in agreement with previous observations [8]. After this, R_q_ continued to decrease but at a much slower rate due to different wear mechanisms. For a large part of the experiment, the surfaces can be considered to be stable in terms of their geometry.

**Fig. 7:**
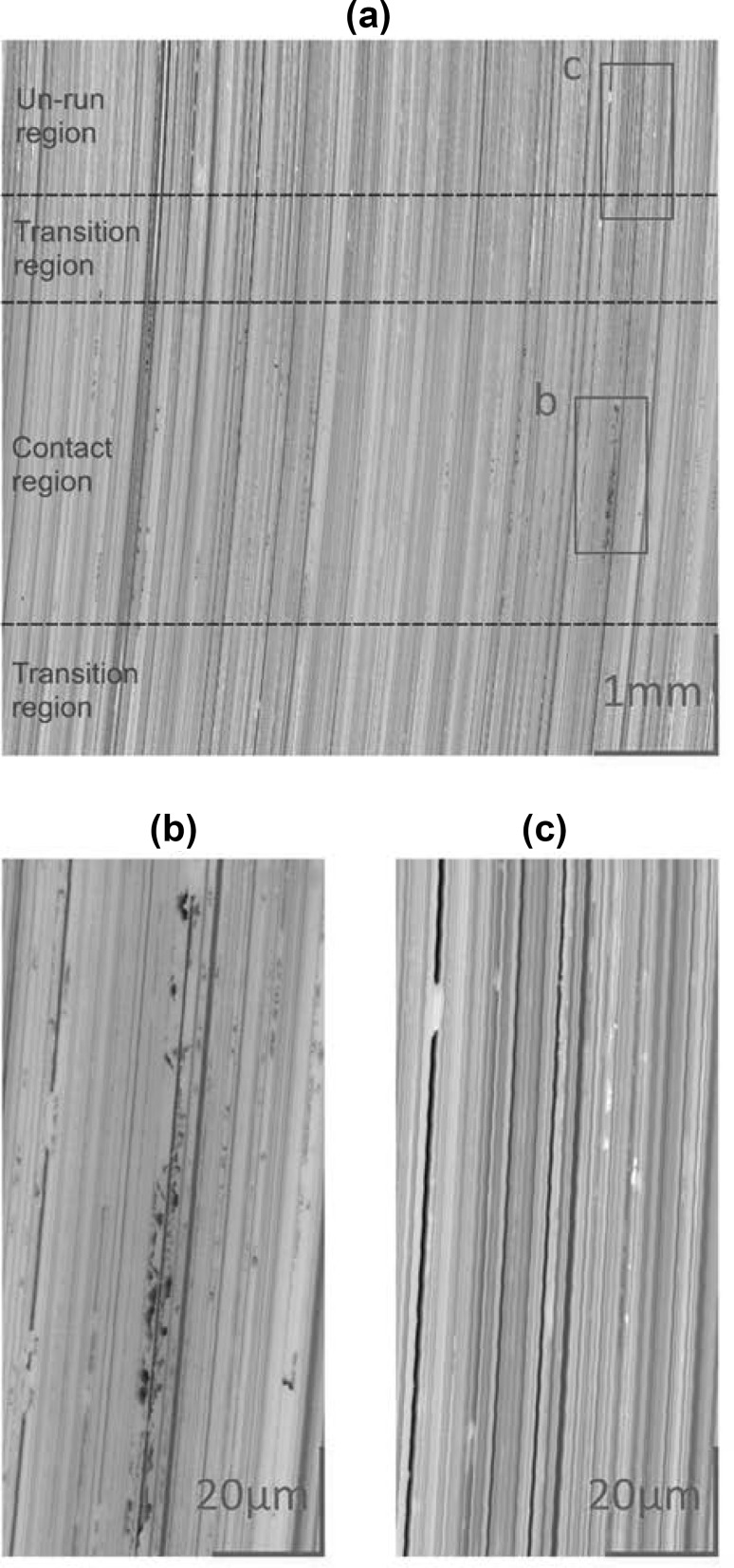
**a** 3D roughness measurement of the fast disk taken at the end of the test, valleys are dark, peaks are light. **b** Close-up of the wear track showing a prominent series of micro-pits, visible as dark speckles, aligned along a section of profile. **c** Close-up of the profile section outside of the contact area for comparison

Figure 7 shows the 3D roughness of the fast disk at the end of the test, after 2.5 million cycles. Micro-pits were found across the entire width of the contact area and tended to be clustered along the positions of previously prominent asperities. These observations are largely consistent with previous work [8]; however, since this experiment was designed specifically for comparison with numerical fatigue calculations, it was run under constant operating conditions to simplify comparisons, compared to the previous work where micropitting was the outcome of a complex series of tests under a wide range of operating conditions. Further discussion of the detailed evolution of surface profiles during the test may be found in the Fatigue Modelling section.

## Fatigue Modelling and Comparison with Experimental Results

**Fig. 8:**
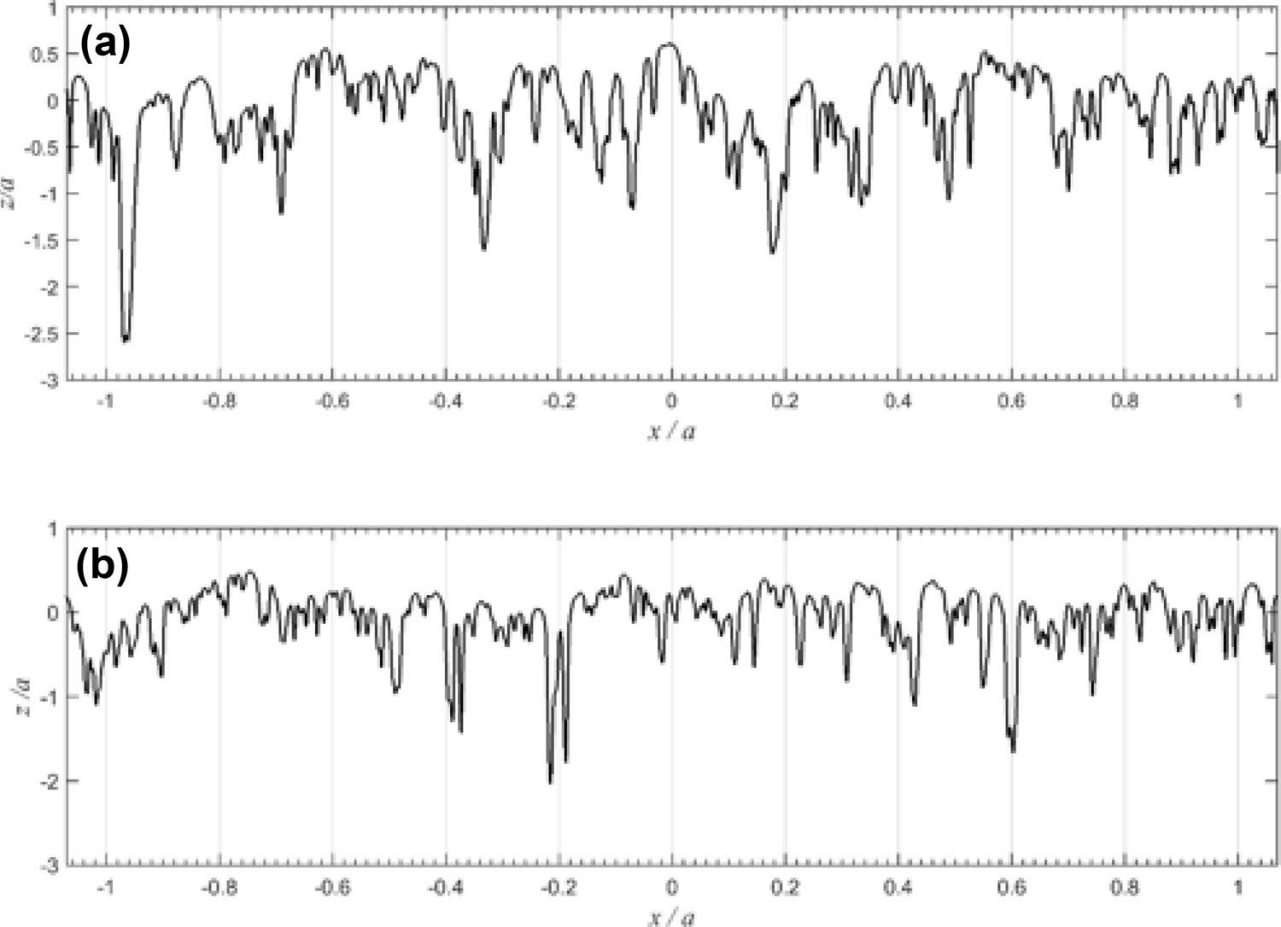
Roughness profile for **a** slow and **b** fast disks, with metal below the trace

The sections of disk profiles considered for this analysis are shown in Fig. 8. These were taken from the profiles measured following the initial running-in load stage, LS1, given that previous work [8] has shown that the majority of surface modification which affects micro-EHL behaviour occurs during the first few cycles of operation.

These profiles were repeated to create multiprofiles as previously described, and were then used in micro-EHL simulations using the experimental conditions shown in Table 2, with simulation parameters given in Table 3, where the lubricant properties correspond to the mean steady-state temperature of the test disks measured during the experiment, 88 °C.

**Table 3:** Operating conditions used in the micro-EHL simulation

Fast surface peripheral velocity(ms^− 1^)	3.99
Slow surface peripheral velocity(ms^−1^)	2.39
Lubricant viscosity (Pas)	0.0112
Pressure viscosity coefficient (Pa^−1^)	1.44 × 10^−8^
Eyring shear stress (MPa)	10.0
Young’s modulus (GPa)	207
Poisson’s ratio	0.3

**Fig. 9:**
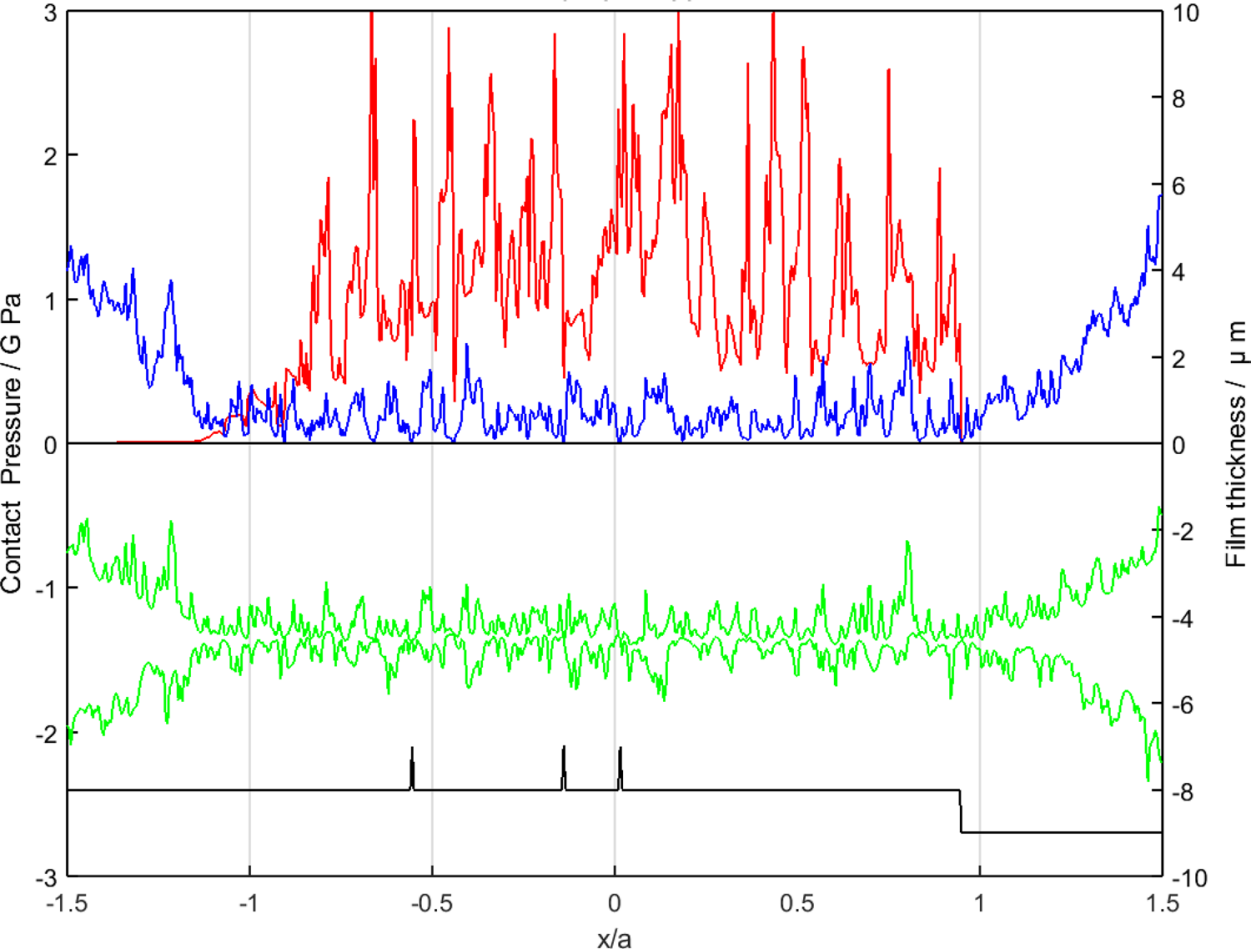
A particular time step during the micro-EHL simulation: pressure distribution (red curve), film thickness distribution (blue curve), in-contact cavitation (black curve) and deflected surface profiles (green curves offset for clarity). (Color figure online)

Figure 9 shows the pressure, film thickness distribution and deflected rough surface profiles at a single time step during a transient simulation of the two roughness profiles. This is an example time step when both surfaces within the contact are fully rough. The deflected surface profiles are offset for clarity so that the relative magnitudes of the surface roughness and the film thickness can be appreciated. The pressure at asperity contacts is clearly much higher than the Hertzian value and in this time step high extreme pressure spikes of 3, 2.8 and 3.25 GPa occur at positions *x*/*a* = − 0.625, 0, 0.5, respectively, where the prominent surface asperities can be seen to be in close interaction. The black curve indicates the contact condition at each point in the mesh for the time step. It has three levels, corresponding to cavitated film (lower level), full film (central level) and direct contact (upper level). In Fig. 9, there are three occurrences of direct asperity contact of x/a = − 0.56, x/a = − 0.125 and x/a = 0.0. It is clear that the pressure values in the valley zones are much lower compared with pressure at the asperities but, generally, the pressurised valley features contribute significantly to the load carrying of the overall EHL film. The contact is operating well within the mixed lubrication regime and has a nominal *Λ* ratio of 0.5.

**Fig. 10:**
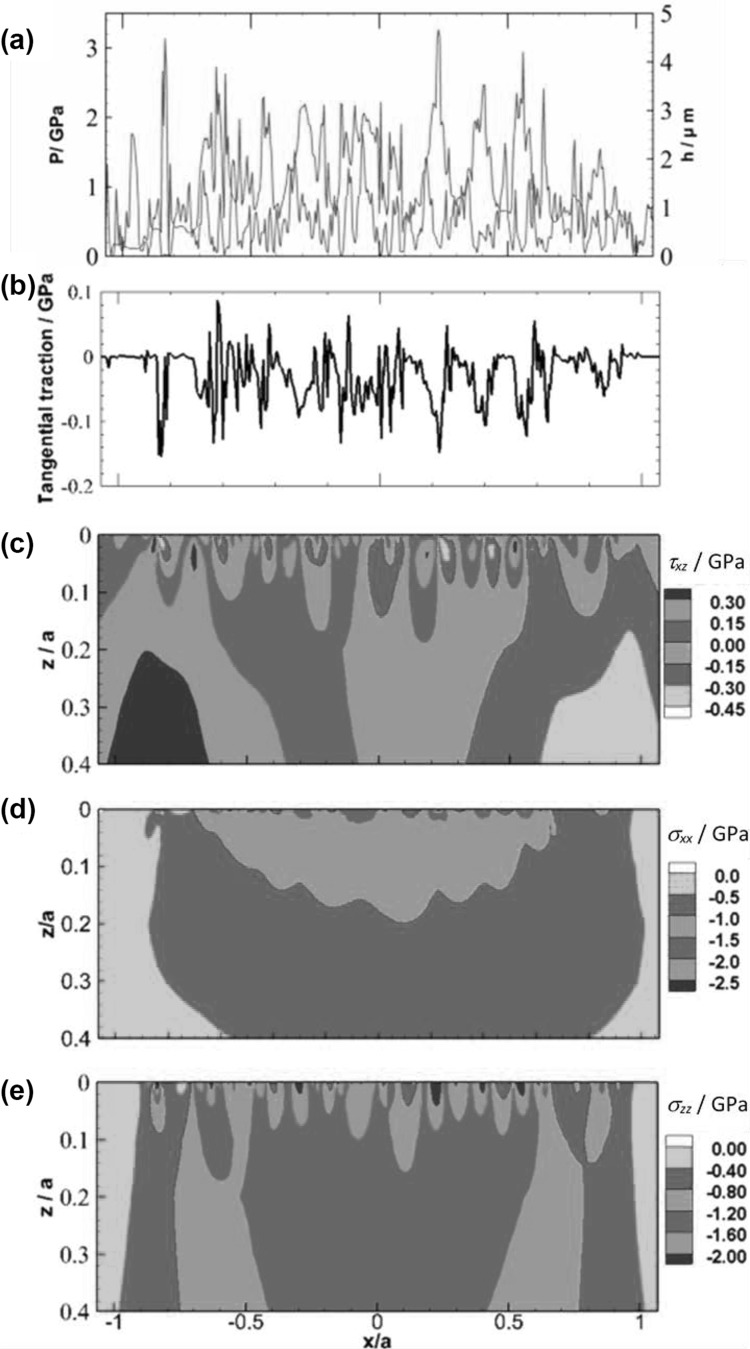
Model predictions at an example time step of the transient analysis of the slow surface. **a** contact pressure (red) and film thickness (blue), **b** tangential surface shear stress, and stress fields of **c** *τ*_*xz*_, **d** σ_*xx*_, and **e** σ_*zz*_. (Color figure online)

Figure 10 shows the transient EHL solutions, as well as the corresponding components of the transient sub-surface stress fields for an example time step within the EHL analysis. Figure 10a shows the pressure and film thickness, and Fig. 10b shows the surface shear stress acting. Figure 10c, d, e show the stress components which exhibit high stress concentrations over areas with 0 ≤ z/a ≤0.1. The *σ*_*zz*_ component is negative everywhere as the contacting bodies are under compression.

These stress concentrations occur during the short times that asperity features are in close proximity and cause intense cyclic loading that may lead to fatigue damage in the form of surface and subsurface micropitting and surface crack formation.

**Fig. 11:**
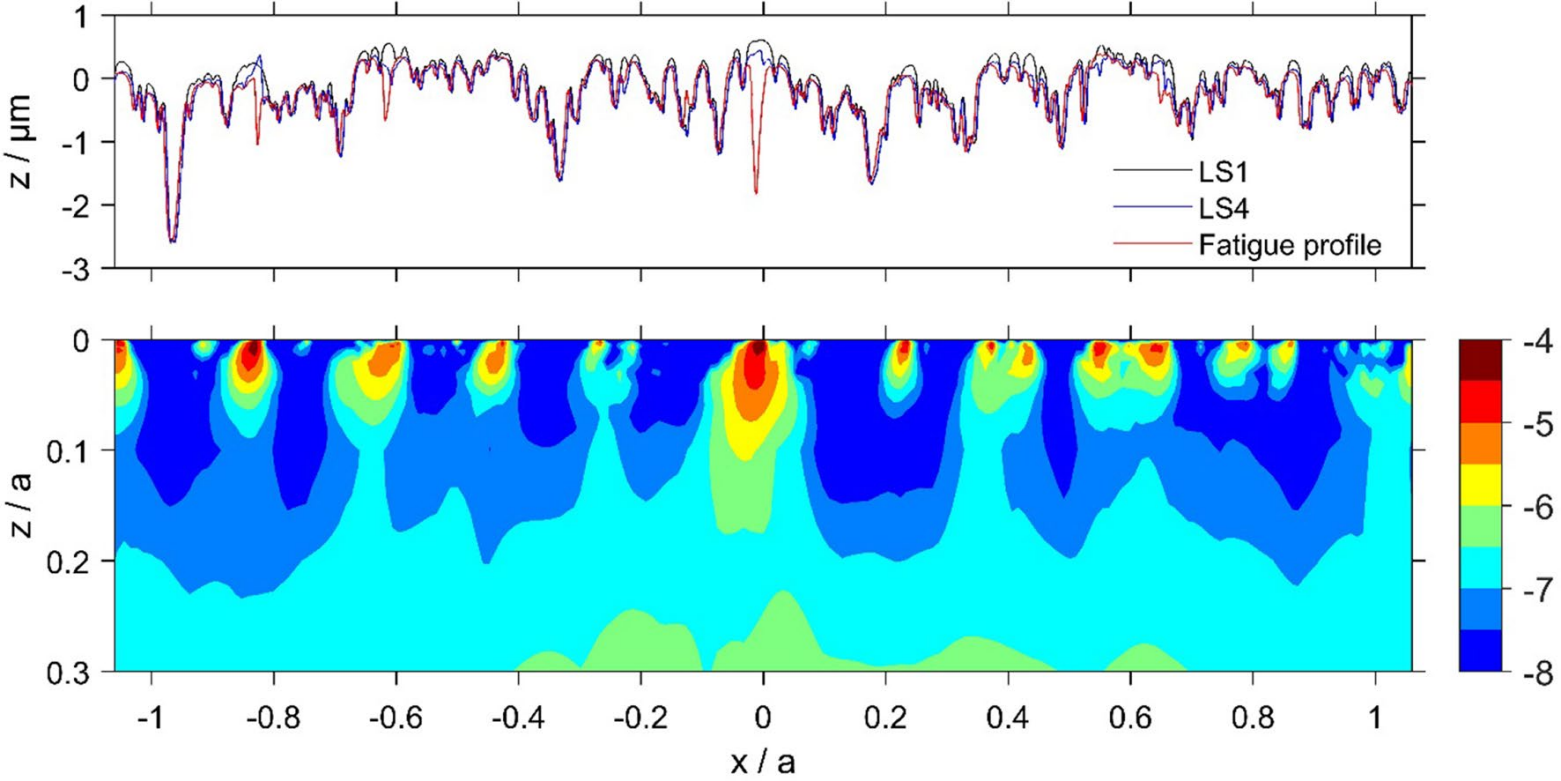
Slow disk relocated profiles (upper frame) and contours of log_10_ (*D*) using Fatemi and Socie criterion (lower frame), where accumulated damage, *D* = 10^−n^, indicates fatigue in 10^*n*^ cycles

**Fig. 12:**
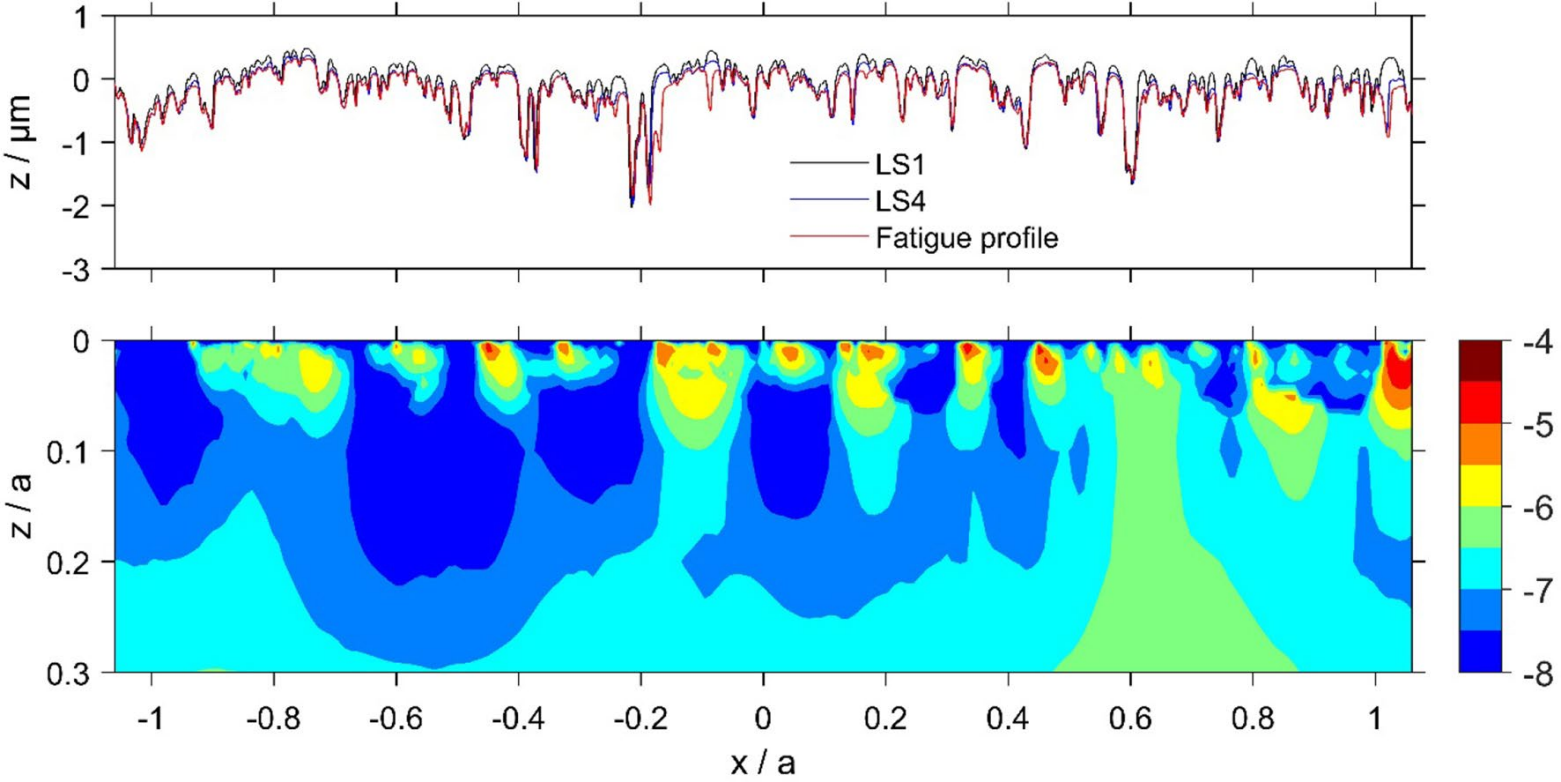
Fast disk relocated profiles (upper frame) and contours of log_10_ (*D*) using Fatemi and Socie criterion (lower frame), where accumulated damage, *D* = 10^−n^, indicates fatigue in 10^*n*^ cycles

The calculated accumulated damage value varies over the 2*a* ⋅ *a* section of material and its variation is presented as contour plots for the sub-surface volume of the slow and fast disks, as shown in Figs. 11 and 12. The roughness profiles, which are seen in the upper part of Figs. 11 and 12, termed as Load Stage 1, Load Stage 4 and Fatigue Profile, were taken from the pitting experiment. These correspond to approximately 3000 fast disk cycles and 1800 slow disk cycles for the Load Stage 1 profiles; 200,000 fast disk cycles and 124,000 slow disk cycles for the Load Stage 4 profiles; and 1 million fast disk cycles and 0.6 million slow disk cycles for the Fatigued Profile.

Comparison of the Load Stage 1 and Load Stage 4 traces shows minimal further surface modification following the running-in changes which occurred during Load Stage 1. The roughness profiles obtained at each stage were aligned in the profile trace direction to minimise the positional error between deep valley features.

**Fig. 13:**
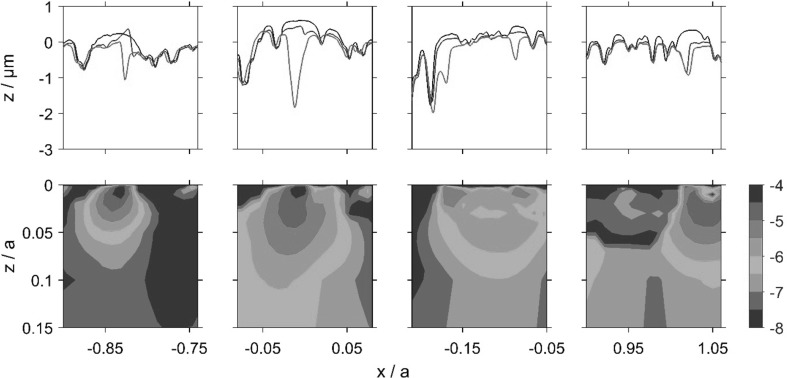
Relocated profiles showing micropit initiation (upper frame) and corresponding near-surface contours of log_10_ (*D*) using Fatemi and Socie criterion (lower frame). The first two examples (from the left) are from Fig. 11 and the others from Fig. 12

Comparison of the roughness profiles in Figs. 11 and 12 shows that some peaks had been replaced with small valley features by the time that the Load Stage 4 profiles were taken. This is evidence of initiation of micropitting occurring between Load Stages 1 and 4. Examples of this can be seen in Fig. 11 at *x*/*a* values of − 0.8,− 0.6, 0, 0.44 and 0.64, and in Fig. 12 at *x*/*a* = − 0.28, − 0.24, − 0.08, 0.79 and 1.04. Comparison with the Fatigued Profiles shows that further micropits have been formed or enlarged in the further running after the Load Stage 4 measurements were taken. Figure 13 shows four enlarged sections of the profiles where micropits have developed and these can be seen to initiate between Load Stages 1 and 4, clearly demonstrating regions of high damage immediately sub-surface associated with micropit initiation.

**Fig. 14:**
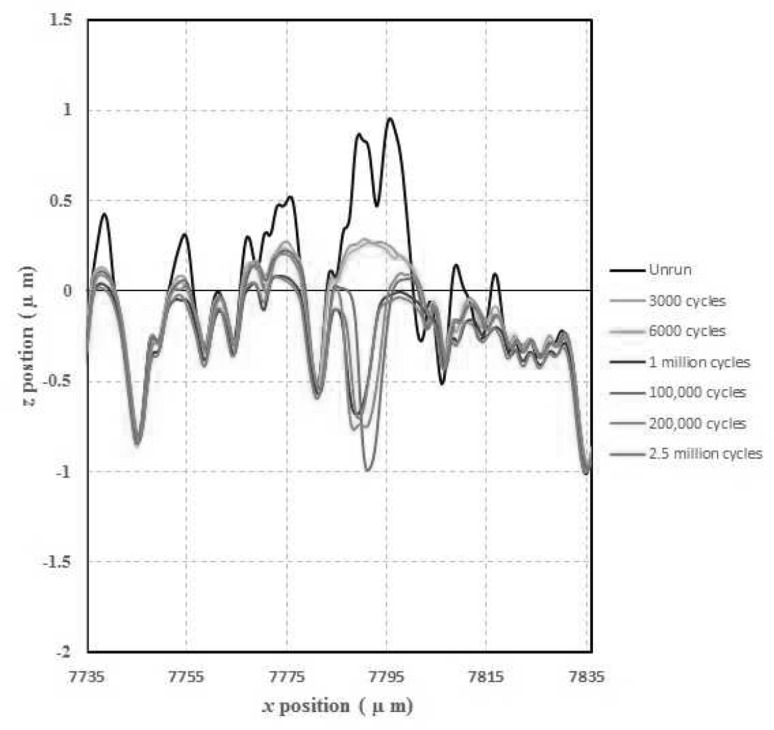
Relocated fast disk profiles showing formation of a micropit

Further detailed analysis of the formation of micropits was carried out using the relocated profiles obtained after each load stage during the experiment. Figure 14 shows the evolution of a micropit located on the fast surface, with the original as manufactured surface presented together with those measured after 3000, 6000, 100,000, 200,000, 1 million and 2.5 million cycles.

It is clear from Fig. 14 that the surfaces initially undergo some running-in, which takes the form of plastic deformation, and the previously aggressive asperities become flattened and more rounded after only 3000 operating cycles. By 100,000 cycles, the large asperity at around *x* = 7790 µm has been removed and a pit has formed, which remains relatively stable until the end of the experiment. Although the measured depth of the pit does appear to fluctuate, it must be remembered that these profiles are measured in-situ on the test rig. This means that there will inherently be some axial location error in these measurements, particularly as the means of cleaning the disks prior to measurements being taken is unavoidably less thorough than the ultrasonic bath that is used when the disks are removed from the rig.

At this point, it is relevant to consider the potential links between running-in and subsequent initiation of micropits. Previous work by the authors [7] has clearly demonstrated the effects of plastic deformation of asperities during running-in on the residual stress field immediately sub-surface. It was found that the yielded material is surrounded by a ring of tensile stresses, which reach the asperity surface with values of the order of the yield stress. The presence of such stresses at the surface is likely to be conducive to the formation and growth of the cracks which lead to micropits. Other workers [9, 33] have linked plastic deformation with localised microstructural changes and subsequent micropitting observed at those positions.

**Fig. 15:**
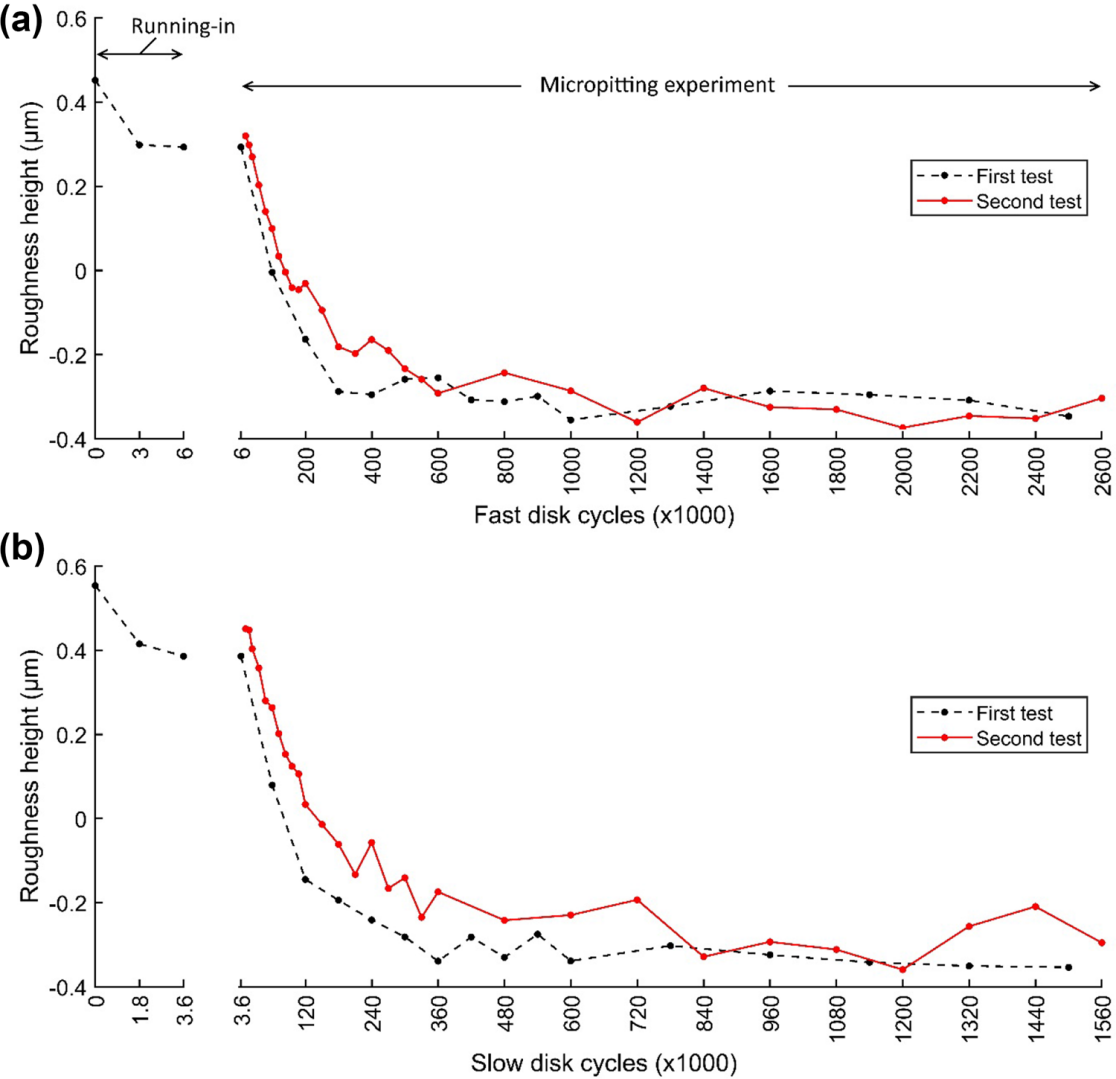
Evolution of mean roughness height for pitted regions of the profiles measured during the micropitting experiments. **a** and **b** refer to the fast and slow disks, respectively

The complete set of 12 mm long centre-line traces taken from both slow and fast disks was then analysed systematically. By comparing realigned profiles with the stable 6000 cycles run-in profile, it was possible to identify regions where large changes in profile height occurred during the experiment. This was achieved by evaluating the variance of the group of profile heights at each point which reliably identified regions of the profile where micropits had occurred. The mean profile height of the pitted regions was then plotted against disk operating cycles for all of the load stage measurements and can be seen in Fig. 15a, b for the fast and slow disks, respectively. Figure 15 also includes data from a second micropitting test undertaken to assess repeatability using a new pair of disks. This test was identical to the first test in terms of operating conditions and disk roughness. It did not include detailed running-in measurements, but profile measurements were taken at shorter intervals over the first 200,000 fast disk cycles (10 measurements), and thence at 50,000 fast disk cycle intervals until 600,000 cycles, at which point measurements were taken every 200,000 cycles until the end of the test.

This demonstrates that asperities reduce in height slightly during the running-in stage, but that the significant changes in height as micropits form occurs during the first two post running-in load stages for the original experiment. This is in the period from around 6000 cycles to approximately 200,000 cycles for both disks. The overall trend of these results is for the pitted regions to become deeper as the tests progress to their conclusion after 2.5 million fast disk cycles, but the data at higher cumulative cycles appears noisy. This is likely to be due to the difficulties previously discussed associated with in-situ profilometry. Similarly, the changes in height of non-pitted locations were evaluated and found to be minimal, with a maximum change over the duration of the experiment of less than 0.1 microns. This change is significantly less than the changes experienced by pitted regions, and is thought to be associated with the inherent alignment errors associated with the relocation profilometry technique.

These detailed observations are in good agreement with the contour damage results presented in Figs. 11 and 12. It is clear that the asperities sustain calculated damage levels that are significantly higher than those experienced by the surrounding material. In these figures, the contour levels are chosen to vary logarithmically in value as the difference in D between the areas having the most and least calculated fatigue damage is up to five orders of magnitude. The vertical (*z*) scale of the damage contour is shown as a fraction of the corresponding Hertzian contact semi-dimension, *a*. It can be seen from these figures that the accumulated damage calculated is concentrated near the surface of the prominent asperities features within the approximate range 0.0 < *z*/*a* < 0.07 and this damage occurs after a relatively modest number of load cycles. The results of the damage accumulation model indicate a life between 10,000 and 32,000 cycles for the brown contour areas in Figs. 11 and 12, and between 32,000 and 100,000 cycles for the red contour area. The model results are, therefore, of the same order as the observations, indicating damage initiation occurring early during the micropitting test between Load Stages 1 and 4. This is encouraging given that the model is for two representative run-in surface profiles that in all probability did not run against each other in the actual micropitting test.

Comparing the accumulated damage between the faster and slower surfaces, it is clear that the calculated damage to the slower surface is larger than that on the faster surface. This happens because asperities on the slower moving surface are subject to higher numbers of asperity interactions and physical loading cycles during their passage through the EHL contact than are those of the faster moving surface. Furthermore, it can be seen that a significant amount of plastic deformation occurs during the first load stage of both disks. The asperity features become almost uniformly rounded. It must also be remembered that profile data is simply a snapshot in time of the surface profile—it does not indicate whether asperities are harbouring growing cracks but have not yet become detached from their parent surfaces.

## Conclusions

- This paper gives predictions of micropitting initiation using a shear strain driven fatigue model based on a micro-EHL analysis of the contact of real, run-in rough surfaces.
- The micro-EHL model, operating under heavily mixed lubrication conditions, showed that the time dependent pressure experienced at the encounters between run-in surface asperities were far higher (often by a factor of three or more) than the Hertzian smooth-surface pressure.
- Corresponding experimental investigations showed that initial micropits formed during the first 100,000 cycles of operation, which is in broad agreement with the model predictions where the most heavily damaged asperities failed in the first 30,000–100,000 cycles of operation.
- The asperities for the slower surface in a contacting pair generally experience more intense stress cycles in traversing the contact zone than do those of the faster moving surface.

## Data Availability

Information on how to access the data that supports the results presented in this article can be found in the Cardiff University data catalogue at 10.17035/d.2016.0008118727.
